# Digitally Driven Aerosol Jet Printing to Enable Customisable Neuronal Guidance

**DOI:** 10.3389/fcell.2021.722294

**Published:** 2021-08-30

**Authors:** Andrew J. Capel, Matthew A. A. Smith, Silvia Taccola, Maria Pardo-Figuerez, Rowan P. Rimington, Mark P. Lewis, Steven D. R. Christie, Robert W. Kay, Russell A. Harris

**Affiliations:** ^1^School of Sport, Exercise and Health Sciences, Loughborough University, Loughborough, United Kingdom; ^2^Faculty of Engineering and Physical Sciences, School of Mechanical Engineering, University of Leeds, Leeds, United Kingdom; ^3^School of Science, Loughborough University, Loughborough, United Kingdom

**Keywords:** direct write, aerosol jet printing, microfabrication, neuronal alignment, tissue engineering, biomaterials

## Abstract

Digitally driven manufacturing technologies such as aerosol jet printing (AJP) can make a significant contribution to enabling new capabilities in the field of tissue engineering disease modeling and drug screening. AJP is an emerging non-contact and mask-less printing process which has distinct advantages over other patterning technologies as it offers versatile, high-resolution, direct-write deposition of a variety of materials on planar and non-planar surfaces. This research demonstrates the ability of AJP to print digitally controlled patterns that influence neuronal guidance. These consist of patterned poly(3,4-ethylenedioxythiophene)-poly(styrenesulfonate) (PEDOT:PSS) tracks on both glass and poly(potassium 3-sulfopropyl methacrylate) (PKSPMA) coated glass surfaces, promoting selective adhesion of SH-SY5Y neuroblastoma cells. The cell attractive patterns had a maximum height ≥0.2 μm, width and half height ≥15 μm, Ra = 3.5 nm, and RMS = 4.1. The developed biocompatible PEDOT:PSS ink was shown to promote adhesion, growth and differentiation of SH-SY5Y neuronal cells. SH-SY5Y cells cultured directly onto these features exhibited increased nuclei and neuronal alignment on both substrates. In addition, the cell adhesion to the substrate was selective when cultured onto the PKSPMA surfaces resulting in a highly organized neural pattern. This demonstrated the ability to rapidly and flexibly realize intricate and accurate cell patterns by a computer controlled process.

## Introduction

Within the pharmaceutical sector it is estimated <10% of drugs entering phase I clinical trials proceed to market, with the average cost ranging from $0.8b to $1.7b (Takebe et al., 2018). Consequently, there is a requirement for cost effective biological test platforms that accurately recreate human physiology and can rapidly eliminate ineffective or toxic therapeutics, also known as a “fail-fast” approach (Mohs and Greig, 2017). Traditional 2D cell cultures, utilizing homogeneous inert substrates such as glass or tissue culture plastics, are often used to rapidly screen novel treatments but fail to recreate the complex multi-cellular environments seen within the body (Mirbagheri et al., 2019). One strategy to overcome this limitation is the selective deposition of microscale topographical and chemical cues on homogeneous 2D cell culturing platforms (Bettinger et al., 2009; Mirbagheri et al., 2019; Zhang K. et al., 2019; Shi et al., 2020). These highly structured surfaces composed of various patterns, also known as 2.5 dimensional objects, have been recognized as a promising tool for controlling cells behavior and function, including attachment, migration, proliferation, and differentiation, with cell morphological and functional responses greatly depending on the cell type as well as the pattern type and dimensions (Mirbagheri et al., 2019). As such, these topographical substrates are capable of better mimicking the natural extracellular matrix and have shown great promise as *in vitro* experimental test platforms for applications within both drug discovery and disease modeling in which tissue models should adequately recapitulate the complexity of natural cell organizations (Bettinger et al., 2009; Mirbagheri et al., 2019; Zhang K. et al., 2019; Shi et al., 2020).

A significant challenge in developing these complex micro-environments is the availability of effective and efficient manufacturing processes that allow patterned multi-functional surfaces to be generated (Shick et al., 2019). The specific manufacturing challenges associated with the development of these environments necessitates manufacturing processes that can generate complex micro-topographies, with the capability to rapidly iterate designs, a useful tool when undertaking bioengineered model development (Zhang S. et al., 2019). There is also an increasing requirement for these environments to be biologically active, whereby the cellular environment created is capable of significantly enhancing cellular development (Akhmanova et al., 2015; Janson and Putnam, 2015). This increased functionality can be achieved through both chemical (e.g., polymer brushes) and biological (e.g., growth factors) modifications, supplemented by topographical patterning, multi-material manufacture, and chemical gradients (Liu and Wang, 2014; Koçer and Jonkheijm, 2018). Finally, the manufacture of surfaces from biomimetic molecules such as collagen, fibrin or laminin allows more accurate recreation of *in vivo* biology (Caliari and Burdick, 2016).

Manufacturing processes including photolithography (Karp et al., 2006; Nie and Kumacheva, 2008), soft lithography (Qin et al., 2010; Hardelauf et al., 2014; Filipponi et al., 2016), electrospinning (Denchai et al., 2018), and direct-write techniques (Roth et al., 2004; Lind et al., 2017; Suzuki and Teramoto, 2017; Derakhshanfar et al., 2018; Tse and Smith, 2018; Garma et al., 2019; Yuk et al., 2020) have been used to date for the production of patterned bio-interfaces. However, template driven processes such as photolithography and micro-contact printing do not effectively support mass customization and iterative development of novel material formulations. Alternatively, direct-write techniques such as inkjet and extrusion-based printing, are often limited by material selection and offer limited printing resolution, with a minimum feature size of ∼50 μm (Murphy and Atala, 2014). Having the manufacturing capability to rapidly screen a wider range of biomaterials at a smaller scale with high design flexibility, would facilitate the rapid and iterative development of complex *in vitro* environments.

**FIGURE 1:**
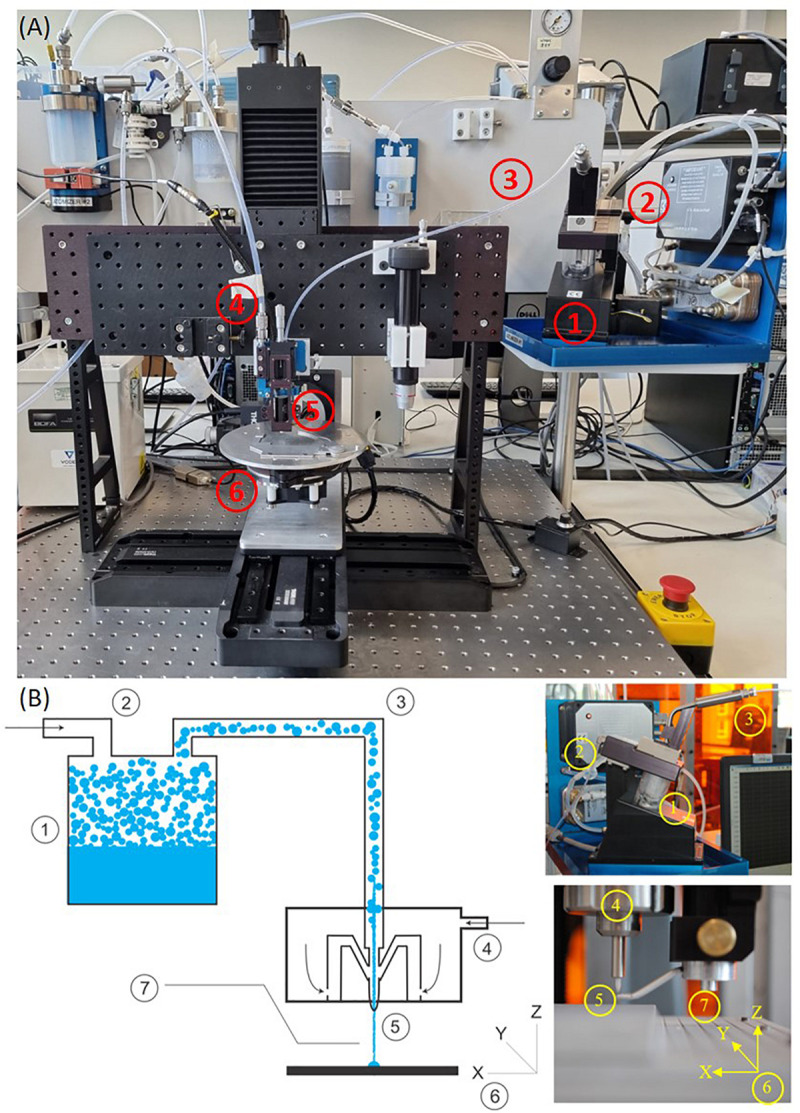
A picture of the machine **(A)** and the description **(B)** of AJP technology. (1) A liquid sample is atomized. (2) An inert gas is used to increase the pressure in the atomizer chamber. (3) The aerosol is transported to the deposition head with the gas acting as a sheath around the aerosol. (4) The aerosol is focused and accelerated by a further annular sheath of inert gas. (5) The resulting high velocity jet is deposited onto the substrate. (6) The automated stage is moved to produce a pattern. (7) On/off patterning is achieved by interrupting the jet with a mechanical shutter.

This research demonstrates the use of Aerosol Jet Printing (AJP) as an enabling manufacturing process which could facilitate the next generation of bespoke *in vitro* environments. Primarily developed for the manufacture of electronic circuitry, AJP is a promising direct write technology that has been applied to a diverse range of applications, including active and passive electronic components, actuators, sensors, as well as a variety of selective chemical and biological responses (Murphy and Atala, 2014; Wilkinson et al.,2019a,b). Within this framework, a comprehensive review on the AJP process and the current state-of-the-art from an applications perspective has been recently published by Wilkinson et al. (2019b). AJP uses a focussed aerosol for the discrete deposition of a wide range of materials with micron scale resolution (∼10 μm) onto a variety of flat and three-dimensional surfaces at nozzle–substrate offsets of 1-5 mm (Grunwald et al., 2010; Cai et al., 2016; Mashayekhi et al., 2016; Secor, 2018). The material is atomized using either focussed ultrasonic energy, or pneumatic shearing of the fluid, depending on the viscosity of the liquid ink, and the required throughput of material for the application. Figure 1A illustrates our AJP apparatus, comprising of a high resolution 5-axis stage which moves the substrate below the aerosol stream under Computer Numerical Control (CNC). An overview of the AJP apparatus and process is illustrated in Figure 1B. AJP has been applied to print a diverse range of materials including polymers, metal nanoparticles, ceramics, and proteins (De Silva et al., 2006; Holthaus and Rezwan, 2008; Grunwald et al., 2010; Maiwald et al., 2010; Hegge et al., 2011; Tamari et al., 2014; Rahman et al., 2016; Zare Bidoky and Frisbie, 2016; Phuah et al., 2020; Williams et al., 2020).

In the field of bioprinting, the use of AJP as a direct-write technique capable of high-resolution deposition of biological materials for cell patterning have been reported by De Silva et al. (2006), and Grunwald et al. (2010). More recently Phuah et al. (2020) have demonstrated biological function of gelatin from AJP lines printed with a minimum width of 30 μm. These promising outcomes further illustrate the use of AJP in cell patterning, microarray and lab-on-a-chip applications, demonstrating that, compared to some bioprinting techniques, AJP offers the capacity to print a wider range of ink viscosities and at a higher spatial resolution. Although AJP has primarily been used for surface patterning, researchers are beginning to explore its potential for producing 3D microstructures with complex architectures (Saleh et al., 2017). Moreover, multi-material aerosol jet printing has been successfully demonstrated for the printing of multilayer and composite structures (Wang et al., 2016; Wilkinson et al., 2019b). Such capabilities illustrate the possible value of AJP in this field to enable a route for the development of devices with increased performance by harnessing the unique capabilities of the process.

In this work, poly(3,4-ethylenedioxythiophene):poly(styrene sulfonate) (PEDOT:PSS) was selected to investigate AJP for micro-scale deposition to create bespoke neuronal culture environments. Among the materials that promote cell attachment, the polymer PEDOT:PSS has received considerable attention because of its unique set of properties. The excellent biocompatibility, the superior flexibility compared to inorganic conductors, and the mixed ionic-electronic conductivity, which provides enhanced communication between cells and devices, make it an amenable interface with biological tissues (Owens and Malliaras, 2010; Higgins et al., 2012; Wang et al., 2016; Ohayon et al., 2017). In addition, PEDOT:PSS-based substrates have been shown to allow the direct electrical stimulation of electrogenic cells (e.g., neurons and muscle cells), regulating or inducing several biological functions (Gomez et al., 2007; Lundin et al., 2011). Such electrical stimulation is not the subject of this current work but its use in this founding research may permit such future extensions on the future. The use of AJP to reliably produce micro-scale printed structures in the region of 20 μm wide from PEDOT:PSS has already been successfully demonstrated by our group (Smith et al., 2018). In this present work, this material was deposited onto both planar glass substrates as well as poly(potassium 3-sulfopropyl methacrylate) (PKSPMA) polymer brush coated glass. PKSPMA polymer brushes have previously been shown to be non-toxic toward the SH-SY5Y neuroblastoma, and due to their hydrophilicity and charged surfaces shown to inhibit cellular adhesion (Pardo-Figuerez et al., 2018). As such when combined with a secondary material that promotes cellular adhesion this polymer is an ideal selection for creating pre-determined regions of neuronal patterning. This pairing of functional materials to create complex small-scale patterns was investigated to provide a new route to model substrates representing variations of *in vivo* neural environments. During this research, the SH-SY5Y human neuroblastoma cell line was utilized, due to complexities of sourcing primary human motor neurons for experimentation. SH-SY5Y cells are a heterogeneous neuron like cell population, capable of differentiating and propagating neurites following exposure to differentiation media. This cell line also has the ability to display the dopaminergic, adrenergic and cholinergic markers typical of *in vivo* neural biology (Pahlman et al., 1984; Kume et al., 2008; Xie et al., 2010). Without precise control over the features of the culture substrate, *in vitro* neuronal cultures are characterized by chaotic environments with unpredictable neuronal directionality. Recreation of organized network structures, and consequently cellular functionality, is therefore a key step to begin building an *in vitro* model that mimics the neuronal circuits found *in vivo*, increasing the applicability of the models toward investigation of the cellular and molecular mechanisms regulating health and disease of the human nervous system.

## Materials and Methods

### Materials

The chemicals (3-aminopropyl)-triethoxysilane (APTES, >98%), triethylamine (TEA, >99.5%), 2-bromoisobutyryl bromide (BIBB, >98%), copper(I) bromide (CuBr, 99.999%), copper(II) bromide (CuBr_2_, 98%), 2,2′-bipyridyl (Bpy, 99%), 3-sulfopropyl methacrylate potassium salt (KSPMA, 98%), (3-glycidyloxypropyl)trimethoxysilane (GOPS, >98%), tetrahydrofuran (THF, >99%), methanol (MeOH, >99.8%), acetone [(CH_3_)_2_C = O] and ethylene glycol [(CH_2_OH)_2_, >99.8%] were purchased from Sigma-Aldrich (Sigma, United Kingdom). PEDOT:PSS (Clevios PH1000^TM^) was purchased from Heraeus Holding (Germany). Glass slides (1.0–1.2 mm thick in dimensions of 25.4 mm × 76.2 mm) were purchased from Scientific Glass Laboratories Ltd. (United Kingdom). For cell culture experiments, Dulbecco’s Modified Eagle Medium (DMEM) GlutaMAX, heat inactivated fetal bovine serum (FBS), Penicillin/Streptomycin (P/S, 10,000 units penicillin and 10 mg streptomycin/mL), alamarBlue^®^ viability assay kit, 4′,6-diamidino-2-phenylindole (DAPI) nuclei counterstain and Alexa Fluor^®^ 488 goat anti-mouse IgG were purchased from Fisher Scientific (United Kingdom). Primary antibody monoclonal anti β tubulin III produced in mouse (∼2.0 mg/mL) was acquired from Sigma. All chemicals and reagents were used as per the manufacturer’s instructions.

### Substrate Preparation for Aerosol Jet Printing

#### Glass Substrates

Glass slides were washed in acetone, methanol and distilled water (dH_2_0) and exposed to oxygen plasma (Inseto PE-25) at 75W and a 15 cm^3^ oxygen flow rate for 120 s to remove surface contaminants before the aerosol jet process.

#### PKSPMA Polymer Brush Coated Glass Substrates

Glass slides were washed in acetone, methanol and dH_2_0 before being cleaned for 15 min with a UV/O_3_ cleaner (PR-100, UVP). Cleaned glass slides were placed in a vacuum oven along with 10 drops of APTES on aluminum foil. The vacuum was activated and the glass slides were exposed to the APTES vapor for 30 min at room temperature (RT). Samples were then placed in an oven for 30 min at 110°C. APTES-functionalized samples were placed in dry tubes and purged for 1–2 min with N_2_. The following reagents were then added by syringe: dry THF (10 mL), dry TEA (0.3 mL, 0.21 g, and 2.1 mmol) and BIBB (0.26 mL, 0.48 g, and 2.10 mmol). The tubes were then N_2_ purged for an hour with the samples immersed in this solution at 25°C. Samples were taken out and rinsed with THF, methanol and dH_2_0, and then dried under a flow of N_2_. For the polymer brush attachment, PKSPMA (17.29g and 70.2 mmol) methanol and dH_2_0 (2:1 v/v) were stirred and degassed by bubbling through N_2_ for 20 min in a 100 mL sealed three-neck round bottom flask. After 10 min the monomer was dissolved and 2, 2′-bipyridyne (0.651 g and 4.17 mmol) and copper(II) bromide (0.0179 g and 0.08 mmol) were added. The mixture was stirred and degassed for an hour at 25°C, followed by the addition of copper(I) bromide (0.230 g and 1.6 mmol). Following this step the monomer solution was transferred into tubes containing the initiator coated samples. A N_2_ filled balloon was added to maintain an inert atmosphere for 24 h at 25°C. After the polymerization was complete, the samples were washed sequentially with methanol and dH_2_0, and dried under N_2_.

### Machine Set Up, Material Formulation and Aerosol Processing Parameters

An Optomec Aerosol Jet print engine was engineered into a programmable 5-axis Cartesian stage controlled through a control code (G-Code) input to Mach3 CNC software, which moves the substrate below the aerosol stream with micrometer scale precision. N_2_ was used as the inert sheath and atomizer gas. This apparatus is illustrated in Figure 1. For the initial determination of a suitable print formulation a chemical composition experiment was conducted with: PEDOT:PSS (0.52–1.04%), Ethylene Glycol (0–20%), and dH_2_0 (78.96–99.48%). Full details of the different materials tested are included in the Supplementary Material associated with this document (Supplementary Table 1). Machine processing parameters were varied with this material to optimize the print quality. A 4-way full factorial matrix style DOE was conducted to investigate the effects of; sheath gas flow rate (30-40 standard cubic centimeters per minute, sccm), atomizer gas flow rate (20-25 sccm), print platform scanning speed (70–90 mm/min) and nozzle height from platform (2.5–3.5 mm). The nozzle size was fixed at 100 μm and the atomizing current was kept constant at 0.65 mA. The surrounding environmental factors were closely controlled to minimize the effect on the final material deposition. The specific material and process combinations are included in the Supplementary Table 3.

### Substrate Characterization

Phase contrast microscope images were taken using a LEICA DMIL microscope with a 20× magnification objective. White light interferometry measurements were taken using a 3D Optical Profilometer Zygo NewView 5000. Cross sectional data was analyzed for the maximum height, width at the base of the profile, width at half height and cross-sectional area. Atomic force microscopy measurements were taken using a Veeco Explorer to characterize maximum height, width at the base of the profile, width at half height and surface roughness.

### Substrate Preparation for Biocompatibility Screening

Glass slides were cut into rectangles of an area of 2.5 cm^2^, washed in acetone, methanol and dH_2_0 and then cleaned with a UV/O_3_ photo reactor (PR-100, UVP). Samples were each UV/O_3_ treated for 15 min. Slides were then coated with a 400 μL solution containing varying concentrations of PEDOT:PSS, GOPS, ethylene glycol and dH_2_0 (Table 1). These samples were then baked in an oven at 150°C for 15 min to remove any solvent, leaving a film of the PEDOT:PSS material adhered to the surface of the glass.

**TABLE 1:** Print formulations used to determine biocompatibility.

Formulation	PEDOT:PSS concentration (v/v%)	Ethylene glycol concentration (v/v%)	dH_2_0 concentration (v/v%)	GOPS concentration (v/v%)
1	5	30	61	4
2	5	30	63	2
3	5	30	64	1

### Cell Culture Methodology

#### Neuronal Culture Procedures

The SH-SY5Y neuroblastoma cell line (ECACC) was cultured in growth media (GM) consisting of DMEM GlutaMAX supplemented with 10% v/v heat inactivated FBS and 1% v/v P/S. Cells were incubated in an humid environment of 5% CO_2_ atmosphere at 37°C until they reached an 80% confluency. Cells were treated with trypsin enzyme for detachment, counted and plated evenly onto the patterned samples at a density of 1 × 10^4^ cells/cm^2^. These samples were cultured in GM for a period of 24 h and thereafter, neuronal differentiation was induced by incubating cells in differentiation media (DM), consisting of DMEM GlutaMAX, 10% heat inactivated FBS, 1% P/S and 10 μM of retinoic acid (RA) differentiating agent for another 72 h. All experimental conditions were compared against untreated glass slides. Patterned samples were then fixed for immunocytochemistry. Prior to experimentation, patterned samples were sterilized by incubation in 70% ethanol, and then left to dry under sterile conditions in a biological safety cabinet, followed by an hour period of UV irradiation.

#### PEDOT:PSS Biocompatibility Experimental Treatments

PEDOT:PSS coated glass samples were used to evaluate the direct (*n* = 3) and indirect (*n* = 3) biocompatibility of each polymer per repeat (*n* = 2). To assess direct biocompatibility, 1 × 10^4^ cells were seeded directly onto the samples. Control wells (CON, non-patterned glass) were also seeded at 1 × 10^4^ cells per well in 2 mL GM. To assess the in-direct biocompatibility of chemical leachate from each substrate, coated glass samples were placed in acellular wells, containing only medium. Chemically leached medium was then transferred to its corresponding experimental well during each repeat, ensuring that cellular medium was directly representative of either cumulative sample degradation or CON at each specific time-point. Each media only well was pre-incubated with 2 mL GM for 24 h prior to commencing each experiment, to ensure cells were seeded within media that had been exposed to the chemical leachate of each sample. Medium, once transferred to experimental wells, was replenished for further 24 h incubation prior to each subsequent media change. During the last 24 h in which the cells were cultured in GM, DM was added to the media only wells in preparation to induce differentiation in cell cultures.

#### Immunocytochemistry

Cells were fixed with 3.7% formaldehyde in phosphate buffered saline solution (PBS) and subsequently blocked and permeablised with 5% goat serum (GS) and 0.2% Triton-X100 in tris-buffered saline (TBS, pH 8.5) for 30 min at RT. Thereafter, a solution with monoclonal mouse anti-β-tubulin III (1:200) antibody and 2% GS in TBS was added and incubated for 2 h. This solution was removed and AlexaFluor488^®^-conjugated goat anti mouse IgG (1:200) was added along with nuclear counter stain DAPI (1:1000), containing 2% GS and 0.2% Triton-X100 in TBS. The solution was incubated in the dark for an hour and thereafter, samples were rinsed with TBS and mounted onto cover slides using Fluoromount^TM^ Aqueous Mounting Medium.

#### Image and Statistical Analysis

Fluorescence microscope images were taken using a LEICA DM2500 fluorescence microscope with a 20× magnification objective. Each experimental condition consisted of a minimum of 2 substrates per technical (biological) repeat, with a minimum of 3 technical repeats per condition. Five random images were taken at the end of the experimental repeat for every sample at every line width, totaling a minimum of 30 images analyzed per condition. Nuclei and neurite alignment were obtained using a customized Fiji macro plugin. The angle of the nuclei/neurite was obtained by using the channels as the reference point. Consequently, vertical lines parallel to the line edges were considered as the point where cells were perfectly aligned (0 degrees). The nuclei/neurite alignment was then cumulatively expressed by interquartile ranges. Statistical analyses and significance of data were determined using IBM^©^ SPSS^©^ Statistics version 23. All the data is presented as mean (±standard deviation), derived from the means of each of the 6 unique substrates (minimum value, 3 technical repeats). Statistical differences between data were measured via a one way ANOVA followed by a Tukey’s *post hoc* test. Differences were considered statistically significant for *p*≤0.05.

## Results and Discussion

### Development of a PEDOT:PSS Material Formulation for Aerosol Jet Printing

Different examples of inks based around commercial formulations of PEDOT:PSS containing a co-solvent regime of dH_2_0 and ethylene glycol can be found in literature for printing with AJP (Ha et al., 2010, 2013; Kim et al., 2013; Aga et al., 2014; Hong et al.,2014a,b; Zare Bidoky and Frisbie, 2016). In this work, Clevios^TM^ PH1000 formulation, a commercially available aqueous dispersion of PEDOT:PSS with a 1.0–1.3% solid content and a viscosity of 15-50 cP (as reported by the supplier H.C. Starck, Leverkusen, Germany) was employed. Using PH1000 as the functional material, a methodology was established for testing the dilution ratios of the two co-solvents (water and ethylene glycol) for AJP to highlight the importance of the initial solvent mixture and define a suitable PEDOT:PSS ink to use during subsequent processing. As a 10% by volume dilution using ethylene glycol is frequently used in the literature (Ha et al., 2010, 2013; Kim et al., 2013; Hong et al.,2014a,b) the upper and lower limits in this experiment were set to 0 and 20% by weight to test around an already proven formulation. The nine material formulations are generated at defined locations between the upper and lower concentration bounds and are reported in Supplementary Table 1. The corresponding printed lines were observed by optical microscopy and evaluated in terms of line uniformity, overspray, homogeneity and feature size. Representative images of the printed lines obtained using each formulation are included in the Supplementary Material attached to this document (Supplementary Figure 1) and the observations of each result are summarized in Supplementary Table 2. From this analysis it was evident that formulation P4 (1.04% v/v PEDOT:PSS, 20% v/v ethylene glycol, 78.96% v/v dH_2_0), generated the most uniform line, with minimal overspray and also a narrow line width (Supplementary Figure 2). For this reason, it was selected for further investigation.

### Characterization of Machine Processing Parameters to Generate Micro-Features

Producing suitable aerosol-jet printed micro-scale features with the ultrasonic atomizer requires precise control over key processing variables, such as the atomization frequency, the flow rate of the carrier gas that transports the aerosol mist to the substrate (carrier flow), the flow rate of the sheath gas that collimates the aerosol into a narrow beam (sheath flow), nozzle diameter, the speed of the substrate under the beam (stage speed), and the distance between the substrate and the nozzle (working distance). The relationship between adjustable process parameters and the geometry of the aerosol-jet printed lines has been documented previously by Mahajan et al. (2013) in the case of printing silver nanoparticles; within this framework, they evidenced that the critical factors affecting silver track size are the ratio of the sheath and carrier gas flow rates (defined as the focusing ratio) and the stage speed. In particular, they reported that the line width decreases with increasing the focusing ratio and stage speed. Simultaneously, the thickness increases with increasing the focusing ratio but decreases with increasing stage speed. These results were also confirmed in our early studies on the aerosol-jet printing of PEDOT:PSS micro-features (Smith et al., 2018). Moreover, previous results from our group defined the processing conditions to reliably print PEDOT:PSS lines in the region of 20 μm wide (100 μm diameter nozzle, atomizing current 0.65 mA, sheath flow 40 sccm, carrier flow 20 sccm, stage speed 70 mm/min, working distance 3 mm) (Smith et al., 2018). Subtle variations in these key processing parameters can have a significant impact on the rate of material deposition and ultimately the precise geometries deposited onto the substrate.

Starting from the above mentioned previous studies (Mahajan et al., 2013; Smith et al., 2018), the generation of precise micro-features of PEDOT:PSS (formulation P4, 1.04% v/v PEDOT:PSS, 20% v/v ethylene glycol, 78.96% v/v dH_2_0) on glass substrates via AJP was informed by a full factorial design of experimentation (DoE) assessing the relative importance of four variables on PEDOT:PSS track profile: sheath flow, carrier flow, working distance and stage speed (see section “Materials and Methods” and Supplementary Table 3 for details).

White light interferometry was used to assess the cross sectional area, width at base, width at half height and the step height of the sixteen different PEDOT:PSS tracks deposited via AJP onto glass substrates (see “Materials and Methods” section for details). The results for the full DoE are reported in Supplementary Table 4. The PEDOT:PSS tracks printed in these trials showed a width at half height ranging from 20 to 40 μm, a width at base from 30 to 50 μm, and a cross sectional area from 5.5 to 12 μm^2^.

The results of the analysis of the sensitivity of the process to individual variables are displayed in Supplementary Table 5. The analysis of two and multi factors interactions are available, respectively, in Supplementary Tables 6, 7. These results confirmed that cross sectional area is reduced by increasing sheath gas from 30 to 40 sccm, reducing carrier gas from 25 to 20 sccm, and increasing stage speed from 70 to 90 sccm, and identified the sheath and carrier flow ratio as the primary influence on track profile. The effect of varying the working distance, and the effect of two and multi factor interactions were negligible in comparison. In conclusion, to make large changes to the line geometry, the carrier gas should be changed first. Once a geometry in a suitable region has been achieved, it should be tuned by changes to the sheath gas flow rate and the stage speed.

### Printing Micro-Features for SH-SY5Y Cells Alignment

The analysis of the interactions reported in the previous paragraph informed the settings of processing parameters for the application of PEDOT:PSS aerosol jet printed micro-features to be used to guide neuronal cell culturing. Since previous literature has demonstrated increased SH-SY5Y neurite and nuclei alignment for cells confined within patterned substrates with a width <100 μm (Nam et al., 2014), we focused on suitable processing parameters to manufacture track sizes of 15, 20, 30, 40, and 50 μm, that would promote significant neuronal alignment. In these experiments, the scanning speed and the working distance were fixed, respectively, at 100 mm/min and 3 mm; for each track size, the corresponding nozzle size, sheath flow and carrier flow are summarized in Table 2. Regarding the nozzle size, previous studies have demonstrated that the printed line width and thickness are also a function of this process parameter, evidencing that finer lines are produced from smaller nozzles (Mahajan et al., 2013). However, as focusing ratio increases, the pressure built up in the nozzle represents a limitation for smaller nozzles, as the maximum pressure is reached at lower focusing ratios when compared with larger nozzles. Except for the line widths <20 μm, almost the entire spectrum of line widths achieved using a 100 μm nozzle can be achieved with a 150 μm nozzle at comparatively higher sheath gas flow rates. The use of larger nozzles presents many advantages compared to the smallest ones; larger nozzles yield a wider range of both line width, sustain higher sheath gas flow rates and reduce the risk of clogging (Mahajan et al., 2013). For these reasons, except for 15 and 20 μm lines, the 150 μm nozzle was used for the rest of the target line width.

**TABLE 2:** Aerosol jet printing (AJP) machine processing parameters used to achieve a range of PEDOT:PSS track widths on a glass substrate.

Target line width (μm)	Nozzle size (μm)	Sheath gas flow rate (sccm)	Carrier gas flow rate (sccm)
15	100	50	18
20	100	50	20
30	150	70	25
40	150	60	25
50	150	50	25

*Scanning speed = 100 mm/min, working distance = 3 mm.*

In order to be employed as a substrate template for neuronal cell culturing, the Aerosol Jet printed PEDOT:PSS patterns must be stable when placed in aqueous physiological conditions for long periods of time without showing delamination or redispersion. Using the above mentioned formulation (formulation P4, 1.04% v/v PEDOT:PSS, 20% v/v ethylene glycol, 78.96% v/v dH_2_0), printed PEDOT:PSS features showed significant degradation when submerged in cell culture media for 72 h, and in some cases complete removal from the glass substrate. To avoid delamination/degradation of the films in aqueous environment, PEDOT:PSS dispersions are typically mixed with other chemical compounds, with notable attention focused toward the silane based cross-linking agent 3-glycidoxypropyltrimethoxysilane (GOPS), that is often adopted to enhance the mechanical stability of PEDOT-based devices on glass and plastic substrates (Zhang et al., 2016; ElMahmoudy et al., 2017). With respect to cell culture applications, while the addition of GOPS promotes PEDOT:PSS adhesion to glass substrates, it must not compromise the biocompatibility of the material. For this reason, the effect of the addition of GOPS adhesion promoter to the previously defined print formulation on the final material biocompatibility was first investigated, and the results are reported as follows.

### Development of a Biocompatible PEDOT:PSS Formulation for Neuronal Culture

**FIGURE 2:**
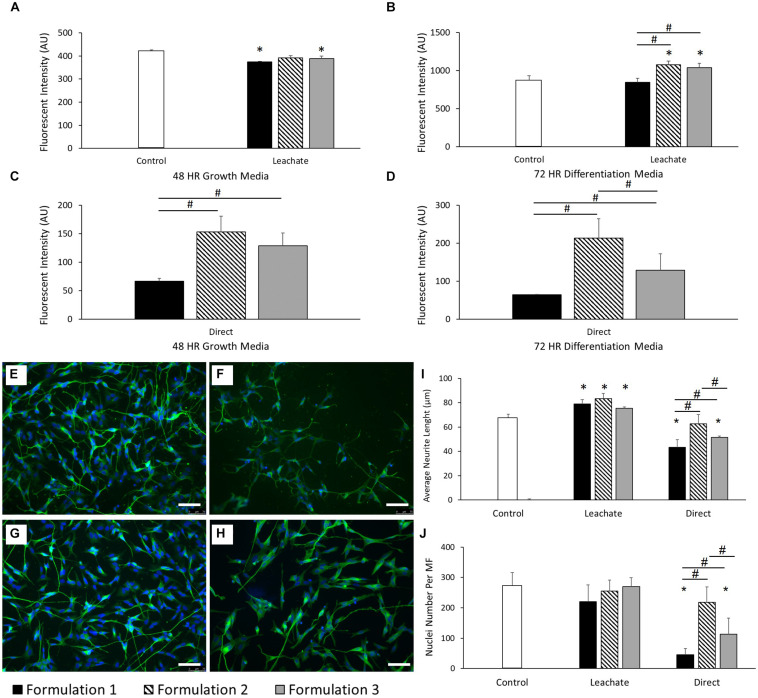
**(A)** Cellular viability of SH-SY5Y neuroblastoma cells cultured in the chemical leachate of PEDOT:PSS coated glass substrates after 48 h in neuronal GM. **(B)** Cellular viability of SH-SY5Y neuroblastoma cells cultured in the chemical leachate of a PEDOT:PSS glass substrates after 72 h in neuronal DM. **(C)** Cellular viability of SH-SY5Y neuroblastoma cells cultured directly onto a PEDOT:PSS glass substrates after 48 h in neuronal GM. **(D)** Cellular viability of SH-SY5Y neuroblastoma cells cultured directly onto a PEDOT:PSS glass substrates after 72 h in neuronal DM. Data presented as mean ± SD from *n* = 6 experimental repeats in each condition. # significantly different within group, * significantly different from control (*P* < 0.05). **(E–H)** Morphological staining of SH-SY5Y neuroblastoma cells cultured directly on PEDOT:PSS coated glass substrates: **(E)** uncoated glass control, **(F)** formulation 1, **(G)** formulation 2, **(H)** Formulation 3. Green = β-tubulin, blue = DAPI. Scale bar = 100 μm. **(I)** Average neurite length of SH-SY5Y neuroblastoma cells after 72 h in DM, cultured in both the chemical leachate of a PEDOT:PSS coated glass substrate and directly onto the material. **(J)** Nuclei number per microscope field (MF, 20× magnification) of SH-SY5Y neuroblastoma cells after 72 h in DM, cultured in both the chemical leachate of a PEDOT:PSS glass substrate and directly onto the material. Data presented as mean ± SD from *n* = 6 experimental repeats in each condition. # significantly different within group, * significantly different from control (*P* < 0.05).

The effect of the addition of GOPS adhesion promoter to the previously defined PEDOT:PSS formulation on the final material biocompatibility was investigated for the SH-SY5Y neuronal cell line (see Table 1 and Cell Culture Methodology details in the “Materials and Methods” section). For all the formulations, there was no visible degradation or peeling after incubation in neuronal GM for 72 h. As evidenced in Figure 2A, SH-SY5Y cells were shown to demonstrate reduced cellular viability when compared to control after 48 h in growth media (GM) when cultured in the chemical leachate from PEDOT:PSS formulation 1 (*P* < 0.05) and formulation 3 (*P* < 0.05). There was no significant difference when comparing between each of these formulations. However, after 72 h in differentiation media (DM), formulation 2 (*P* < 0.0001) and formulation 3 (*P* < 0.05) displayed increased cellular viability compared to control (Figure 2B). When comparing between formulations there was a significant increase in viability shown by formulation 2 (*P* < 0.0001) and formulation 3 (*P* < 0.001), suggesting a cellular preference over formulation 1. This indicates the inclusion of increasing concentrations of the adhesion promotor GOPS to have a negative effect on cellular behavior. This result was confirmed by SH-SY5Y neuronal cells cultured directly onto the materials, which demonstrated a preferential cellular response for formulation 2 (*P* < 0.0001) and formulation 3 (*P* < 0.001) after 48 h in GM (Figure 2C). After 72 h in DM, formulation 2 had a preferential cellular response over both formulation 1 (*P* < 0.0001) and also formulation 3 (*P* < 0.05) (Figure 2D). The viability data for the 3 formulations suggests that each of these biomaterials is a viable culture material for SH-SY5Y when cultured indirectly in the chemical leachate. However, when cultured directly onto these materials then formulation 2 (2% GOPS) is preferential.

Neurite length was also assessed as a morphological indication of SH-SY5Y differentiation on each of the substrates. Figures 2E-H report the morphological staining of SH-SY5Y cells cultured directly on PEDOT:PSS coated glass substrates, respectively, for control (E), formulation 1 (F), formulation 2 (G), and formulation 3 (H). Figure 2I shows that the average neurite length increased significantly across each formulation when compared to control (all *P* < 0.05) after 72 h in DM when cultured in the chemical leachate from each material, without any difference when comparing between formulations. However, there was a reduction in neurite length observed in formulation 1 (*P* < 0.0001) and formulation 3 (*P* < 0.001) when compared to control after 72 h in DM when cultured directly onto the material. There was also a significant increase in neurite length demonstrated by formulation 2 when comparing directly with both formulation 1 (*P* < 0.0001) and formulation 3 (*P* < 0.01). As shown in Figure 2J, no significant difference was observed between the nuclei numbers of each condition after 72 h of culture in the chemical leachate of each material. However, formulations 1 (*P* < 0.0001) and 3 (*P* < 0.0001) demonstrated a reduction in nuclei number when compared to both control, and also formulation 2 (all *P* < 0.05), when cultured directly onto the materials. The significant reductions in morphological behavior demonstrated by formulation 1 discounted the inclusion of this condition in further optimization reported below.

### Topographical Characterization of PEDOT:PSS Micro-Features for SH-SY5Y Cells Alignment on Glass and PKSPMA Coated Glass Substrates

**FIGURE 3:**
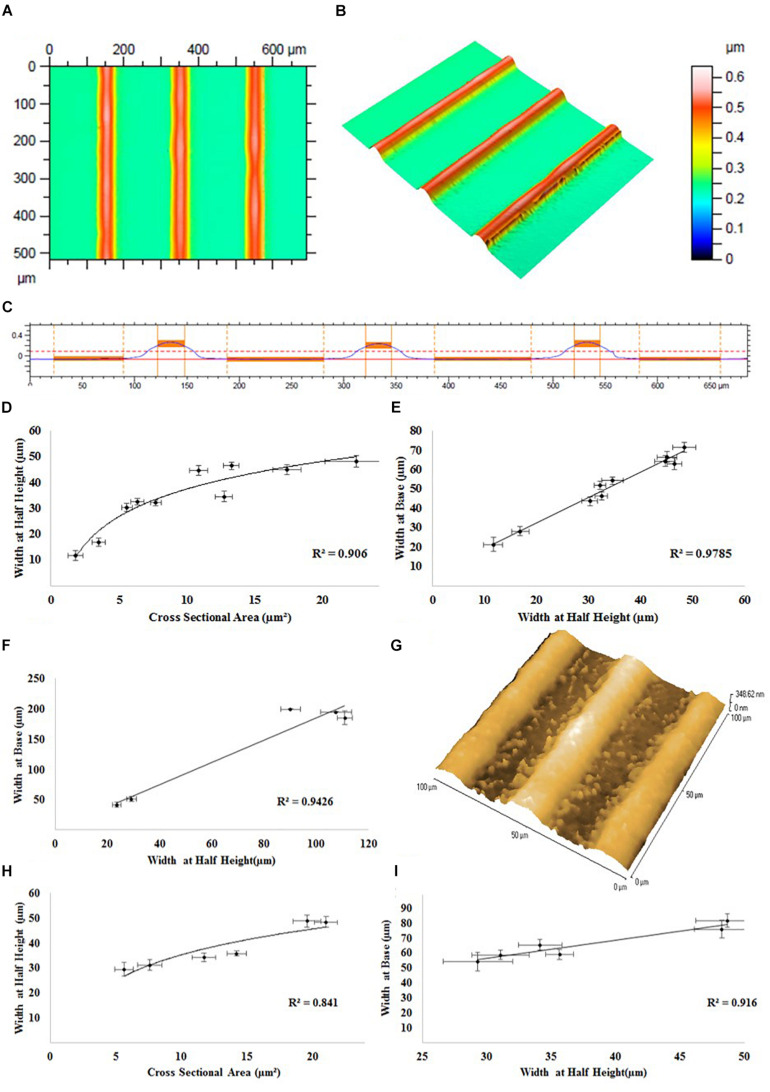
Representative white light interferometry measurements of PEDOT:PSS tracks on a glass substrate: **(A)** 2D top down graphical representation, **(B)** 3D graphical representation, and **(C)** 2D cross sectional graphical representation of a track profile. **(D)** Track width at half height vs. track cross sectional area. **(E)** Track width at half height vs. track width at base. **(F)** Atomic force microscopy measurements of PEDOT:PSS tracks on a glass substrate. **(G)** A representative AFM image of the surface of the tracks. White light interferometry measurements of PEDOT:PSS tracks on a PKSPMA coated glass substrate. **(H)** Track width at half height vs. track cross sectional area. **(I)** Track width at half height vs. track width at base.

The effect of the addition of the adhesion promoter GOPS on the printability of the PEDOT:PSS formulation P4 was investigated, and the remaining formulations (formulation 2 and 3) were tested for aerosol jet printing deposition. These experiments showed that by using higher concentration of GOPS (>1%) the printing quickly starts to become unstable, resulting in non-uniform patterns, until the complete and permanent clogging of the nozzles. Since the clogging of the nozzle did not occur for lower GOPS concentration, formulation 3 was then selected for the printing of PEDOT:PSS micro-features for SH-SY5Y cells alignment, using the printing parameters reported in Table 2. Using this new formulation, there was a noticeable decrease (average 24.2%) in the printed line width at the same process conditions after the addition of the adhesion promotor. This decrease could be accounted for by slightly changing the print parameters so was deemed acceptable. The profile of the printed PEDOT:PSS tracks on glass substrates using this new formulation was analyzed by white light interferometric measurements: a representative example of these measurements is reported in Figure 3, which shows the 2D top down graphical representation (A), the 3D graphical representation (B) and the 2D cross sectional graphical representation (C) of a track profile.

The results of white light interferometry measurements of PEDOT:PSS tracks on glass substrate can be analyzed by directly comparing the geometric parameters of the printed tracks (i.e., cross sectional area, width at half height, track width at base). As evidenced in Figure 3D, a logarithmic relationship (*R*^2^ = 0.906) was observed between track cross sectional area and width at half height, indicating an increase in track width in proportion to the square root of the track area. Equally a linear relationship (*R*^2^ = 0.979) between track width at the base and half height (Figure 3E) indicates that a comparable track profile was generated throughout the experiments, regardless of alterations in print processing parameters. The linear relationship between width at base and width at half height (*R* = 0.9426) was confirmed by further analysis via atomic force microscopy (AFM) (Figure 3F). Roughness measurements from the AFM suggested a smooth surface (Ra < 3.5 nm, RMS < 4.1). Measurements of the track heights were also taken and are included in the Supplementary Material attached to this document (Supplementary Figure 3).

The specifics of the line geometry such as the overall shape, maximum height, width at base, width at half height, side wall angle and surface roughness are subject not only to influences from the material formulation and process parameters, but also material/surface interaction and drying characteristics. White light interferometry was also used to assess deposits of PEDOT:PSS onto a PKSPMA coated glass substrate. A logarithmic relationship (*R*^2^ = 0.841) was observed between track cross sectional area and width at half height, with the profiles achieved typically being highly comparable to those generated on the non-treated glass substrates (Figure 3H). A linear relationship was observed between width at half height and width at the base (*R* = 0.916), once again suggesting a consistent print profile both across the different track sizes and also when comparing between substrates (Figure 3I). The smaller contact angle of water on PKSPMA indicates a more hydrophilic surface than cleaned glass. This resulted in increased spreading of the deposit on the surface resulting in wider lines at the same processing parameters.

The non-linear relationship between cross sectional area and track width, evidenced on both glass (*R*^2^ = 0.906) and the PKSPMA (*R*^2^ = 0.841), is typical of jetting processes, with track widths typically proportional to the square root of the track area. This trend gives rise to an asymptotic relationship between track width and area. The printed tracks proved to be of a uniform geometry independent of material deposition rates or the overall track width. This was confirmed by a strong linear relationship between track width at the base and track width at half height, on both glass (*R*^2^ = 0.979) and the PKSPMA (*R*^2^ = 0.916) coated glass substrate.

### Characterization of Neuronal Alignment on PEDOT:PSS Micro-Features Printed Onto a Glass Substrate and a PKSPMA Polymer Brush Coated Glass Substrate

**FIGURE 4:**
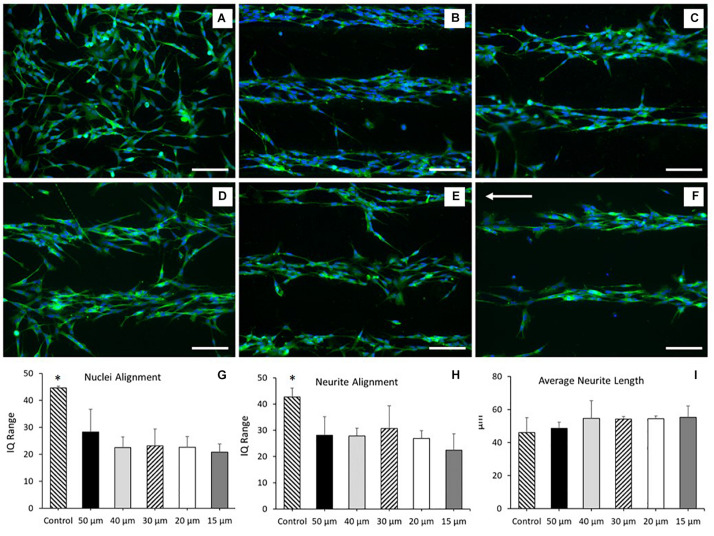
Morphological staining of SH-SY5Y neuroblastoma cells cultured on patterned PEDOT:PSS glass substrates. Green = β-tubulin, blue = DAPI. Arrow indicates direction of PEDOT:PSS tracks deposited via aerosol jetting. **(A)** Untreated glass control. **(B)** 50 μm tracks. **(C)** 40 μm tracks. **(D)** 30 μm tracks. **(E)** 20 μm tracks. **(F)** 15 μm tracks. Scale bar = 100 μm. **(G)** Nuclei alignment of SH-SY5Y neuroblastoma cells after 72 h in DM, cultured directly onto a patterned PEDOT:PSS glass substrate. **(H)** Neurite alignment of SH-SY5Y neuroblastoma cells after 72 h in DM, cultured directly onto a patterned PEDOT:PSS glass substrate. **(I)** Average neurite length of SH-SY5Y neuroblastoma cells after 72 h in DM, cultured directly onto a patterned PEDOT:PSS glass substrate. Data presented as mean ± SD from *n* = 6 experimental repeats in each condition. ^∗^significantly different from all other conditions (*P* < 0.05).

SH-SY5Y cells were cultured onto PEDOT:PSS tracks deposited onto glass substrates via AJP, following the experimental protocol reported in the “Materials and Methods” section. Figures 4A–F reports the morphological staining of SH-SY5Y neuroblastoma cells cultured on patterned PEDOT:PSS glass substrates with different track widths. As shown in Figure 4G, a significant increase in nuclei alignment (all *P* < 0.0001) was demonstrated across each of the track sizes when compared to control. However, there was no increase in alignment when comparing between tracks of differing widths. The same trend was observed for the neurite alignment with a significant increase (all *P* < 0.05) across track sizes against control, but with no change when comparing between tracks of differing widths (Figure 4H). There was however a non-significant reduction in both average nuclei alignment (26.5%) and average neurite alignment (20.3%) when comparing between the 50 and 15 μm tracks. This data indicates a strong cellular response to the topography generated by the AJP printed PEDOT:PSS tracks. There was no statistical significance when comparing average neurite length between all the different substrate conditions indicating no change in cellular differentiation in response to this topography (Figure 4I).

These results indicated that the SH-SY5Y cell line responded to the topographical stimulus generated by the AJP deposited tracks, without significant alteration to the morphology of the cell. However, no statistical variation in neurite or nuclei alignment between each of the track sizes was observed. This suggested that whilst the cells responded to the topographical stimulus generated by the jetted bio-substrate, the addition of a secondary chemical stimulus would be required to facilitate greater cellular alignment.

**FIGURE 5:**
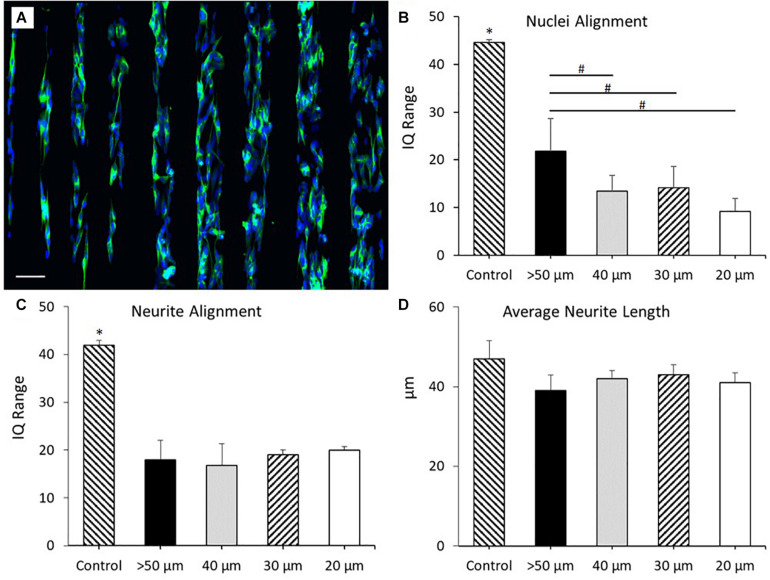
**(A)** Morphological staining of SH-SY5Y neuroblastoma cells cultured on PEDOT:PSS tracks deposited via AJP onto PKSPMA coated glass substrates. Track widths ranging from 20 to 100 μm (left to right). Green = β-tubulin, blue = DAPI. Scale bar = 100 μm. **(B)** Nuclei alignment of SH-SY5Y neuroblastoma cells after 72 h in DM, cultured directly onto a PEDOT:PSS patterned PKSPMA coated glass substrate. **(C)** Neurite alignment of SH-SY5Y neuroblastoma cells after 72 h in DM, cultured directly onto a PEDOT:PSS patterned PKSPMA coated glass substrate. **(D)** Average neurite length of SH-SY5Y neuroblastoma cells after 72 h in DM, cultured directly onto a PEDOT:PSS patterned PKSPMA coated glass substrate. Control condition refers to untreated glass. Data presented as mean ± SD from *n* = 6 experimental repeats in each condition. # significantly different within group, ^∗^significantly different from all other conditions (*P* < 0.05).

Polymer brush coated glass surfaces have previously been utilized to generate bio-substrates that prevent direct neuronal adhesion, but do not inhibit cellular growth and differentiation elsewhere in the culture (Zhou et al., 2013). PKSPMA polymer brush coated glass substrates have previously been shown to demonstrate these properties, making it an ideal candidate as the “repulsive” chemical coating to complement our “attractive” PEDOT:PSS printed tracks. For this reason, experiments were repeated culturing SH-SY5Y cells onto PEDOT:PSS tracks deposited onto PKSPMA coated glass substrates via AJP. Figure 5A report the morphological staining of SH-SY5Y neuroblastoma cells cultured on PEDOT:PSS patterned PKSPMA coated glass substrates with different track widths. Figure 5B demonstrates a significant increase in nuclei alignment (all *P* < 0.0001) across each of the track sizes when compared to control. Likewise, tracks >50 μm in width demonstrated reduced nuclei alignment than all the 40 μm (*P* < 0.05), 30 (*P* < 0.05), and 20 μm tracks (*P* < 0.001). No statistical significance was observed when comparing between the 3 smallest track sizes, however, the most aligned nuclei were observed in cells cultured onto the 20 μm tracks (IQ range = 9.3 ± 2.7), indicating a relationship between reduced track width and cellular alignment (Figure 5C). All track sizes showed increased neurite alignment when compared with control (all *P* < 0.0001), but with no variation when comparing between tracks of different widths. This indicates that whilst the chemical and topographical cues generated by the substrate resulted in increased neurite alignment, the submicron geometries of SH-SY5Y neurites necessitates a further reduction in track width to facilitate further alignment. There was no statistical significance when comparing average neurite length between all the different substrate conditions indicating no change in cellular differentiation in response to this topography (Figure 5D).

These results demonstrated that the addition of the “repulsive” PKSPMA coating significantly increased nuclei and neurite alignment across all track sizes. For the smallest of these track sizes (20 μm) nuclei alignment (IQ range) reduced from 22.5 to 9.3 through the addition of the repulsive coating. This trend was consistent when comparing between each of the track sizes, with the highest IQ range for the coated samples (50 μm = 21.8) being lower than all but the 15 μm uncoated substrates.

### Complex PEDOT:PSS Pattern Design Printed Onto PKSPMA Polymer Brush Coated Glass Substrate

**FIGURE 6:**
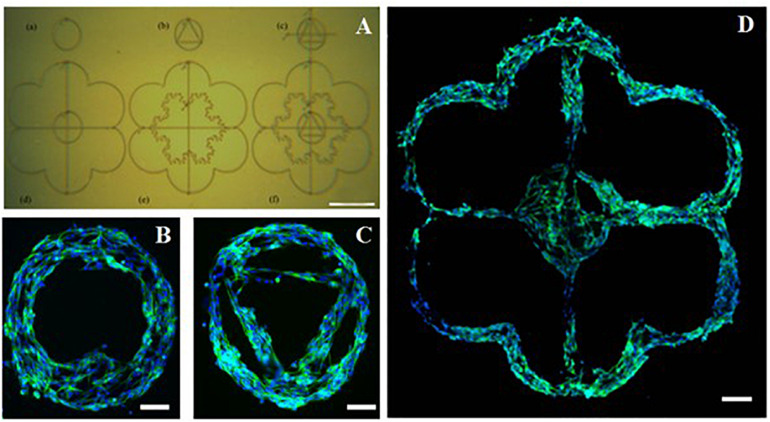
Complex poly(3,4-ethylenedioxythiophene)-poly(styrenesulfonate) (PEDOT:PSS) pattern printed onto a poly(potassium 3-sulfopropyl methacrylate) (PKSPMA) coated glass substrate generated via AJP. **(A)** Patterned surface imaged before cell culture. **(B–D)** Morphological staining of SH-SY5Y neuroblastoma cells cultured on patterned substrate. Green = β-tubulin, blue = DAPI. (Scale bar **(A)** = 500 μm, **(B,C)** = 50 μm, **(D)** = 100μm).

To demonstrate some of the potential of this AJP technique, bespoke freeform patterns were generated using the optimized PEDOT:PSS formulation (Figure 6) and subsequently used to show designed cell culture formations. The individual patterns in this example were printed within a period of between 6 s (pattern A) and 20 s (pattern D). The print time for the entire set of patterns was <90 s. The designs used were selected to illustrate the ease at which increasing complexity could be achieved and to show the design freedom afforded by this technology. Complementary to the processing speed, is also the efficiency of material usage as the material is only printed where required. This material usage is much lower compared to other manufacturing techniques (Lan et al., 2017).

However, when considering the use of AJP there are some other characteristics that should also be considered. One consideration is the speckling or overspray effect which was observed. This results in a small number of particles outlying the edges of the line track, effecting the edge definition (Reitberger et al., 2016). When printing smaller line widths (<20 μm), high sheath or low atomization gas flow rates can result in areas where the line pinches inwards or discontinues (Tait et al., 2015). The ends of lines are regions of unpredictability, as the deceleration of the stage and the machine shutter lead to additional material build-up. The materials and processing definitions are also not well documented for many new materials. This means that in practical terms, whenever a new material is desired, a significant amount of effort must be put into determining the materials suitable printing parameters, and identifying effects that may be specific to the intended application (Agarwala et al., 2017). Most of this definition focusses on line width and surface interactions alone, and currently specifications for height or specific line profiles are not yet commonplace. For this reason, material processing characterization for all new print formulations, such as the data detailed in this research, is important to developing a fuller suite of functional materials for AJP.

## Conclusion

In this work a materials formulation (1.04% v/v PEDOT:PSS, 20% v/v ethylene glycol, 78.96% v/v H_2_O, 1% v/v GOPS) that allowed precise AJP deposition of PEDOT:PSS was developed, capable of producing smooth (Ra < 3.5 nm and RMS < 9 nm) tracks with micro-geometries (widths < 20 μm and depths < 500 nm). The addition of an adhesion promotor (GOPS) allowed these bio-substrates to be exposed to a cell culture environment for a period of 96 h without any feature degradation. These tracks were printed onto both glass and PSKPMA polymer brush coated glass substrates. Subsequently these bio-interfaces were cultured with the SH-SY5Y cell line and shown to induce significant neurite and nuclei alignment within the culture, by generating chemical and topographical cues. We have demonstrated that the process allowed the manufacture of customizable patterns, as well as the use of multiple materials to allow increased substrate functionality. To develop this concept further future work will focus on additional development and characterization of both the bio-substrate, including a comprehensive assessment of bio-substrate electrical properties such as conductivity/impedance within culture mediums, as well as additional characterization of the neuronal population including functional assessment, e.g., live cell calcium imaging, electrical field stimulation, and additional mapping of morphological and genetic development across prolonged experimental time courses. In addition, the application of this technology to biological environments (e.g., ocular, cortical, and/or neuromuscular) whereby controlled cellular development and increased functionality which require complex anisotropic bio-substrates will require additional demonstration.

This manufacturing approach allows the creation of complex 2.5D topographical substrates for *in vitro* cell culture studies while allowing rapid iterative design changes, and so extending the range and diversity of studies that can be explored effectively and economically. The entire design and manufacturing process is digitally driven, thereby providing the capability to rapidly alternate and produce different cell guidance designs, and to do so within time and cost boundaries that would be unachievable by template-based manufacturing approaches. Wider appealing factors of such a digital manufacturing include the ability to remotely receive designs from around the globe to distributed manufacturing locations, thus extending the reach of the research community it may serve. It is suggested that future research could extend capabilities and applications by further developing the range of printable bio-materials, whilst increasing the complexity of design through the generation of multi-material and 3D substrates, opening new possibilities to create realistic *in vitro* cell models. Lastly, but significant to its uptake; this scalable, accurate and reproducible digitally-driven processing technology could enable mass production of these novel substrates, paving the way toward potential industrial translation in a wide variety of practical applications, such as modeling diseases and drug discovery.

## Data Availability Statement

The original contributions generated for this study are included in the article/Supplementary Material, further inquiries can be directed to the corresponding author.

## Author Contributions

MS, ST, RK, and RH contributed to the manufacturing technology and engineering. AC, MP-F, RR, ML, and SC contributed to the biological and chemical science. All authors contributed to the article and approved the submitted version.

## Conflict of Interest

The authors declare that the research was conducted in the absence of any commercial or financial relationships that could be construed as a potential conflict of interest.

## Publisher’s Note

All claims expressed in this article are solely those of the authors and do not necessarily represent those of their affiliated organizations, or those of the publisher, the editors and the reviewers. Any product that may be evaluated in this article, or claim that may be made by its manufacturer, is not guaranteed or endorsed by the publisher.

## Supplementary Material

Supplementary Figure 1 Print formulations P1–P9 utilized during initial material screen. Nozzle size = 200 μm, sheath and atomizer gas flow rates = 50 sccm, nozzle height = 3 mm, stage speed = 5 mm/s. sccm, standard cubic centimeters per minute.

Supplementary Figure 2 Representative microscopic image of print formulation P4 (Scale bar = 200 μm).

Supplementary Figure 3 White light interferometry measurements of maximum height vs. cross sectional area for PEDOT:PSS tracks printed onto a glass substrate via AJP.

Supplementary Table 1 Development of an initial PEDOT:PSS material formulation. Nozzle size = 200 μm, sheath and atomizer gas flow rates = 50 sccm, nozzle height = 3 mm, scanning speed = 5 mm/s. sccm, standard cubic centimeters per minute.

Supplementary Table 2 Observations of print formulations P1–P9 utilized during initial material screen.

Supplementary Table 3 Full factorial design of experiment to determine optimized AJP processing parameters for the deposition of PEDOT:PSS onto a glass substrate. Nozzle size = 100 μm. sccm, standard cubic centimeters per minute. The experimental parameters were run three times, on three separate days with a disassembly and clean of the functional end of the machine after each run. The machine was reassembled before each experiment.

Supplementary Table 4 Full details of the matrix style DoE carried out to optimize the machine processing conditions for the AJP of PEDOT:PSS. In each trial, each of the three lines were assessed three times for each geometrical feature (*n* = 9).

Supplementary Table 5 Analysis of individual variables.

Supplementary Table 6 Analysis of two factor interactions.

Supplementary Table 7 Analysis of three and four factor interactions.
