# Supplementary material for: Digitally Driven Aerosol Jet Printing to Enable Customisable Neuronal Guidance
Source: Front Cell Dev Biol. 2021 Aug 30;9:722294. doi: 10.3389/fcell.2021.722294 (PMC8435718; doi:10.3389/fcell.2021.722294)
Supplement: Supplementary Figure 1 — Print formulations P1–P9 utilized during initial material screen. Nozzle size = 200 μm, sheath and atomizer gas flow rates = 50 sccm, nozzle height = 3 mm, stage speed = 5 mm/s. sccm, standard cubic centimeters per minute. [file Data_Sheet_1.docx]

Supplementary Material

# Supplementary Figures

**Supplementary Figure 1:** Print formulations P1-P9 utilised during initial material screen. Nozzle size = 200μm, sheath and atomiser gas flow rates = 50 sccm, nozzle height = 3mm, stage speed = 5mm/s. sccm = standard cubic centimetres per minute.

**
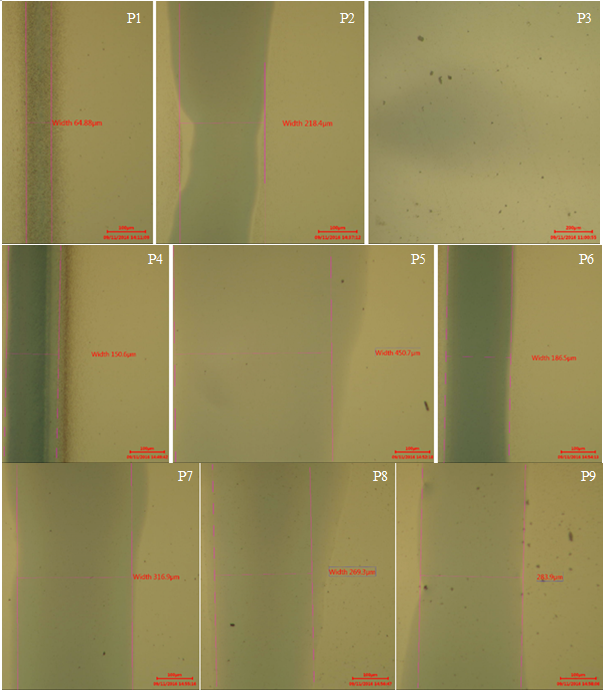
**

**Supplementary Figure 2:** Representative microscopic image of print formulation P4 (Scale bar=200μm).

**
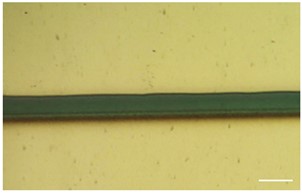
**

**Supplementary Figure 3:** White light interferometry measurements of maximum height vs cross sectional area for PEDOT:PSS tracks printed onto a glass substrate via AJP.


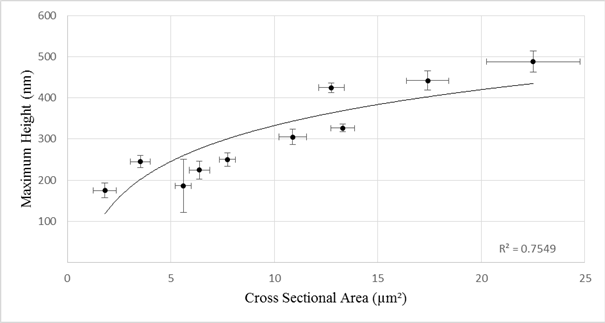


# Supplementary Tables

**Supplementary Table 1:** Development of an initial PEDOT:PSS material formulation. Nozzle size = 200μm, sheath and atomiser gas flow rates = 50 sccm, nozzle height = 3mm, scanning speed = 5mm/s. sccm = standard cubic centimetres per minute.

| **Formulation** | **PEDOT:PSS Concentration (v/v %)** | **Ethylene Glycol**  **Concentration (v/v %)** | **DI Water**  **Concentration (v/v %)** |
| --- | --- | --- | --- |
| P1 | 0.52 | 0 | 99.48 |
| P2 | 1.04 | 0 | 98.96 |
| P3 | 0.52 | 20 | 79.48 |
| **P4** | **1.04** | **20** | **78.96** |
| P5 | 0.52 | 10 | 89.48 |
| P6 | 0.78 | 0 | 99.22 |
| P7 | 1.04 | 10 | 88.96 |
| P8 | 0.78 | 20 | 79.22 |
| P9 | 0.78 | 10 | 89.22 |

**Supplementary Table 2:** Observations of print formulations P1-P9 utilised during initial material screen.

| **Formulation** | **Line Width (µm)** | **Observations** |
| --- | --- | --- |
| P1 | 165 | Incomplete line. Overspray significant even after sintering suggesting under deposition of material. |
| P2 | 219 | Complete line. Significant bulges caused by material wetting, or over deposition. No sign of overspray, and lines width larger than nozzle suggesting over deposition of material. |
| P3 | N/A | No discernible line. |
| **P4** | **150** | **Complete line. Some overspray. No pinching or bulging.** |
| P5 | 450 | Material has spread on substrate causing a very wide line. No sign of overspray as this has likely been covered by material during spreading. Significant bulges likely due to surface tension or over deposition. |
| P6 | 190 | Complete line. Overspray distinguishable on edges of lines. No pinching or bulging. |
| P7 | 317 | Material has spread on substrate causing a very wide line. Evidence of bulging. |
| P8 | 269 | Material has spread on substrate causing a very wide line. Evidence of bulging. |
| P9 | 284 | Material has spread on substrate causing a very wide line. Evidence of bulging. |

**Supplementary Table 3:** Full factorial design of experiment to determine optimised AJP processing parameters for the deposition of PEDOT:PSS onto a glass substrate. Nozzle size = 100μm. sccm = standard cubic centimetres per minute. The experimental parameters were run three times, on three separate days with a disassembly and clean of the functional end of the machine after each run. The machine was reassembled before each experiment.

| **Trials** | **Process Parameters** | | | |
| --- | --- | --- | --- | --- |
|  | **Sheath Flow**  **(sccm)** | **Carrier Flow**  **(sccm)** | **Stage Speed**  **(mm/min)** | **Working Distance**  **(mm)** |
| 1 | 40 | 20 | 70 | 2.5 |
| 2 | 40 | 25 | 70 | 2.5 |
| 3 | 40 | 25 | 90 | 2.5 |
| 4 | 40 | 25 | 90 | 3.5 |
| 5 | 30 | 25 | 90 | 3.5 |
| 6 | 40 | 20 | 90 | 3.5 |
| 7 | 30 | 25 | 70 | 3.5 |
| 8 | 40 | 20 | 90 | 2.5 |
| 9 | 40 | 25 | 70 | 3.5 |
| 10 | 30 | 25 | 90 | 2.5 |
| 11 | 30 | 20 | 90 | 3.5 |
| 12 | 40 | 20 | 70 | 3.5 |
| 13 | 30 | 25 | 70 | 2.5 |
| 14 | 30 | 20 | 90 | 2.5 |
| 15 | 30 | 20 | 70 | 3.5 |
| 16 | 30 | 20 | 70 | 2.5 |

**Supplementary Table 4**: Full details of the matrix style DoE carried out to optimise the machine processing conditions for the AJP of PEDOT:PSS. In each trial, each of the three lines were assessed three times for each geometrical feature (n=9).

| **Process**  **Conditions** | **Results** | | | | | | | |
| --- | --- | --- | --- | --- | --- | --- | --- | --- |
|  | **Step Height (nm)** | | **Width at Half Height (µm)** | | **Width at Base**  **(µm)** | | **Cross Sectional Area**  **(µm^2^)** | |
|  | **Mean** | **SD** | **Mean** | **SD** | **Mean** | **SD** | **Mean** | **SD** |
| 1 | 263 | 35 | 24.6 | 2.7 | 35.3 | 2.7 | 5.95 | 1.03 |
| 2 | 308 | 37 | 32.0 | 2.1 | 40.3 | 2.6 | 9.75 | 1.62 |
| 3 | 297 | 32 | 27.9 | 2.6 | 37.2 | 3.2 | 8.65 | 1.72 |
| 4 | 297 | 40 | 27.7 | 2.2 | 35.4 | 3.0 | 8.06 | 1.50 |
| 5 | 247 | 37 | 33.8 | 6.6 | 45.1 | 6.1 | 9.10 | 2.50 |
| 6 | 256 | 11 | 24.8 | 1.1 | 33.9 | 1.5 | 6.37 | 0.30 |
| 7 | 313 | 23 | 39.2 | 4.0 | 50.2 | 2.4 | 12.10 | 1.07 |
| 8 | 232 | 22 | 21.4 | 2.3 | 31.5 | 2.2 | 5.46 | 0.91 |
| 9 | 293 | 39 | 26.5 | 2.3 | 36.4 | 1.8 | 8.44 | 1.36 |
| 10 | 269 | 15 | 35.4 | 2.1 | 45.5 | 2.4 | 9.71 | 0.72 |
| 11 | 230 | 9 | 27.0 | 1.9 | 35.3 | 2.7 | 6.30 | 0.53 |
| 12 | 252 | 15 | 24.0 | 1.6 | 33.6 | 0.8 | 6.71 | 0.60 |
| 13 | 305 | 43 | 39.8 | 3.0 | 50.8 | 3.9 | 11.93 | 2.47 |
| 14 | 234 | 17 | 26.3 | 1.9 | 36.6 | 2.7 | 6.76 | 0.86 |
| 15 | 272 | 13 | 30.2 | 1.6 | 40.5 | 1.8 | 8.51 | 0.57 |
| 16 | 298 | 13 | 27.2 | 1.1 | 37.2 | 1.8 | 8.29 | 0.57 |

**Supplementary Table 5:** Analysis of individual variables.

| **Effect of** | **Cross Sectional Area (µm^2^)** | **Maximum Height (nm)** | **WHM (µm)** |
| --- | --- | --- | --- |
| Increasing sheath gas | -1.7 | 4 | -6.3 |
| Increasing atomizer gas | 2.9 | 36 | 7.1 |
| Increasing scanning speed | -1.4 | -30 | -2.4 |
| Increasing z stand-off | -0.1 | -6 | -0.2 |

**Supplementary Table 6:** Analysis of two factor interactions.

| Effect of | Cross Sectional Area (µm^2^) | Maximum Height (nm) | WHM (µm) |
| --- | --- | --- | --- |
| Sheath gas/atomiser gas | -0.3 | -2.2 | 11.6 |
| Atomizer gas/scanning speed | -0.3 | -0.7 | 3.1 |
| Scanning speed/z stand-off | -0.1 | 0.7 | 5.1 |
| Sheath gas/atomiser gas | 0.8 | 1.1 | 21.9 |
| Atomizer gas/z stand-off | -0.5 | -1.8 | -1.4 |
| Sheath gas/z stand-off | 0.1 | -0.6 | 5.2 |

**Supplementary Table 7:** Analysis of three and four factor interactions.

| Effect of | Cross Sectional Area (µm^2^) | Maximum Height (nm) | WHM (µm) |
| --- | --- | --- | --- |
| Sheath gas/atomiser gas/z stand-off | -0.4 | -5.5 | -0.3 |
| Sheath gas/atomiser gas/scanning speed | 0.1 | 2 | 0.7 |
| Atomizer gas/scanning speed/z stand-off | 0.1 | -8.9 | 0.3 |
| Sheath gas/scanning speed/z stand-off | 0.3 | 7.2 | 1.6 |
| Sheath gas/atomiser gas/scanning speed/z stand-off | 0.1 | 4.1 | 0 |

**Details on Hadamard matrix approach:**

A Hadamard matrix approach was used to provide an efficient method to reduce the number of tests required to make these comparisons. The Hadamard matrix is simply a list of the sixteen trials that must be carried out to analyse the sensitivity of the process to the four parameters (Supporting Table 3). By pairing the trials in a set order, and comparing the difference between them, several variables and interactions can be analysed in a small number of trials. For example, to examine the effect of turning the sheath flow from low (30 sccm) to high (40 sccm) eight trial pairs were assessed (i.e. 1 and 16; 2 and 13; 3 and 10; 4 and 5; 6 and 11; 8 and 14; 9 and 7; 12 and 15 in Supporting Table 3). In each of these comparisons, only the sheath flow value is different, the other three values are constant. In the same method but using different trial pairs, all variables can be analysed. In addition, trial pairs exist to compare the interactions between two or more variables. Overall, using this method enables one set of results to be analysed for response to all the variables and both two and three factor interactions (Supporting Table 5, 6 and 7).
